# Local Health Department Accreditation Is Associated With Organizational Supports for Evidence-Based Decision Making

**DOI:** 10.3389/fpubh.2019.00374

**Published:** 2019-12-17

**Authors:** Peg Allen, Stephanie Mazzucca, Renee G. Parks, Mackenzie Robinson, Rachel G. Tabak, Ross Brownson

**Affiliations:** ^1^Prevention Research Center, Brown School, Washington University in St. Louis, St. Louis, MO, United States; ^2^Alvin J. Siteman Cancer Center, Washington University School of Medicine, St. Louis, MO, United States; ^3^Department of Surgery, Washington University School of Medicine in St. Louis, St. Louis, MO, United States

**Keywords:** accreditation, evidence-based decision making, evidence-based public health, Governmental health departments, organization

## Abstract

**Introduction:** Recent studies show that health department accreditation from the U.S. Public Health Accreditation Board (PHAB) drives performance management and quality improvement. PHAB standards call for agencies to use evidence in decision making. It is unknown whether accreditation is associated with organizational supports for evidence-based decision making (EBDM). Self-report data from a 2017 survey of U.S. local health departments were analyzed to test relationships of accreditation status with organizational supports for EBDM.

**Methods:** A cross-sectional survey was conducted in this observational study. A total of 579 local health departments were invited to complete an online survey; 350 (60.4%) provided complete data for the present study. The dependent variables were six factors of organizational supports for EBDM previously validated through confirmatory factor analyses. Accreditation status (PHAB-accredited, preparing, not preparing) was the independent variable of interest. Logistic regression analyses controlled for governance (presence of a local board of health; state, local, or shared state and local governance) and jurisdiction population size.

**Results:** PHAB-accredited health departments were more likely to report higher capacity for EBDM, resource availability for EBDM, and evaluation capacity than health departments that reported not yet preparing for accreditation. Health departments that reported preparing for PHAB accreditation showed a non-significant pattern of higher perceived supports for EBDM compared to departments not preparing for accreditation.

**Conclusion:** PHAB standards and the accreditation process may help stimulate health department organizational supports for EBDM.

## Introduction

The U.S. national movement for voluntary public health department accreditation by the Public Health Accreditation Board (PHAB) has gained momentum since the 2011 launch. As of March 29, 2019, 79% of the U.S. population resided in areas served by accredited health departments. The 257 accredited health departments included 218 local, 36 state, and 3 tribal health departments (1). Public health departments prepare for accreditation and reaccreditation by following PHAB Standards and Measures Version 1.5, which calls for documenting use of evidence in practice, completing community health assessments and plans with stakeholders, developing strategic and workforce development plans, and establishing or enhancing quality improvement and performance management systems (2). Agencies are to document that they (1) “identify and use the best available evidence for making informed public health practice decisions” [(2), p. 220]; “promote understanding and use of the current body of research results, evaluations, and evidence-based practices with appropriate audiences” [(2), p. 224].

These accreditation preparations and ongoing activities entail both organizational processes and individual skills in evidence-based decision making (EBDM). EBDM involves using the best available evidence in combination with community preferences to prioritize public health action, plan and implement evidence-based approaches, and conduct sound evaluation (3–5). EBDM is emphasized throughout the PHAB standards (2). For example, EBDM is promoted or required in domains 3, 5, 6, 10, and elsewhere. A literature review identified five domains of organizational supports for EBDM: leadership, organizational climate and culture, workforce development, partnerships, and financial practices (e.g., transparency and outcomes-based contracting) (6). Leadership support for EBDM involves modeling EBDM, envisioning ways to move it forward, creating opportunities for employees to build needed skills for EBDM, ensuring EBDM processes are applied in the day-to-day work, and ensuring best use of limited resources through EBDM (7). Managers supportive of EBDM give staff time to find the evidence and apply the planning and evaluation processes. Through leadership vision and manager expectations and support of EBDM, leaders create an organizational climate and culture in which EBDM is ingrained as the way the organization operates. EBDM involves multiple complex skills, so on-the-job training is important to help employees be able to apply EBDM planning and evaluation processes (3). No one organization can implement the complex policy, system, and environmental changes shown to improve population health, so organizational partners with aligned missions and shared resources are more essential than ever to collaboratively strive to improve public health (6, 8). For individual skill building, on-the-job training opportunities in EBDM now include both in-person and distance learning modalities (9). Despite improvements in some areas, skill gaps persist (10, 11). A 2017 national survey of state and local health department employees found that the highest skill gaps were in financial management, systems and strategic thinking, developing a vision for a healthy community, and change management (11). It remains unknown whether employees in accredited health departments are better equipped and supported to apply EBDM skills than those in non-accredited departments.

The long-term goal of accreditation is to better the population's health through stimulating improved health department performance. There is recent evidence of increased performance management (12–15), quality improvement (12, 13, 16), and planning activities (15) in accredited vs. non-accredited health departments, but little is known about the impact of accreditation on underlying organizational supports for EBDM. The present study starts to address this gap by assessing relationships of accreditation status with organizational supports for EBDM, using primary data from a cross-sectional survey conducted in 2017 with 350 U.S. local health departments (LHDs).

## Methods

The present study was part of a 2017 national survey on organizational supports for EBDM among LHDs (17, 18). Cross-sectional self-report data from an online survey of a sample of U.S. LHDs were analyzed. The institutional review board at Washington University in St. Louis reviewed and approved the study protocol (number 201603157); all participants gave written informed consent in accordance with national guidelines.

### Participant Recruitment

A stratified random sample was selected from eligible LHDs to ensure adequate representation from LHDs of all jurisdiction population size. For each selected LHD, collaborators at NACCHO identified the employee leading chronic disease prevention and control efforts where possible, or the LHD director. One person from each selected LHD was invited to participate. Eligible LHDs had responded to the 2016 NACCHO Profile survey and indicated that the LHD conducted diabetes screening, body mass index screening, or population-based physical activity or nutrition approaches. The chronic disease prevention focus of the eligibility criteria and selection of who to survey in the LHD stemmed from the focus of the study from which these data were drawn, which was on chronic disease prevention. Since we asked about provision of specific evidence-based interventions in community-based chronic disease prevention and control, as well as organizational supports, in our national survey, we deemed the lead chronic disease prevention manager as the person most likely to be knowledgeable in both content areas. In the 2016 National Profile of Local Health Departments survey conducted by NACCHO, 1930 (76.2%) of the invited 2533 U.S. LHDs participated (19). As of the 2016 NACCHO Profile data, 1677 (86.9%) of the 1930 LHDs met the eligibility criteria described above (17). We stratified the sample of 1677 eligible LHDs by small (<50,000), medium (50,000–199,999), and large (≥200,000) jurisdiction population size and then randomly selected 200 LHDs from each of the three strata for a sample of 600 LHDs (17). A total of 579 LHDs were invited after removal of non-valid email addresses.

### Data Collection

The study team used Qualtrics software to collect the self-report online survey data. Participants were invited by email 1 week after a pre-invitation email that described the study purpose. To boost response, non-responding invitees received up to three email reminders and two phone calls or voicemails. Each participant was offered a $20 Amazon gift card at survey completion.

### Measures

#### Survey Development and Testing

The survey was developed from a literature review (6); previous study team surveys; abstraction of additional literature; and three rounds with an expert panel of faculty, study staff, and consultants who had worked in LHDs (17). Ten LHD employees completed cognitive response testing interviews. After refinement, test–retest with 53 LHD participants showed adequate reliability, with most intraclass correlations in the desirable range ≥0.60 (17, 20).

#### Accreditation Status

Accreditation status (independent variable of interest). Self-report data were used to determine accreditation preparation status. Participants were asked “Is your health department accredited or preparing to apply for accreditation through PHAB?” Response options included 1 = “yes, we are currently accredited”; 2 = “yes, recently applied but not yet accredited”; 3 = “yes, but we have not yet applied”; 4 = “no”; 5 = “unsure”. The online PHAB list of accredited health departments was used to confirm PHAB accreditation prior to the date of survey completion.

#### Organizational Supports for EBDM

Organizational supports for EBDM (dependent variables) were assessed in 27 seven-point Likert items with response options 1 = strongly disagree to 7 = strongly agree, with exact item wording shown in Table 1. Although the research team had previously identified organizational support factors as reported by state health department employees, valid factors had not yet been identified for LHDs. Therefore, as described in detail elsewhere, confirmatory factor analyses (CFAs) were conducted in MPlus version 8 with the 27 items to confirm validity of a limited number of factors that would be useful in research and LHD practice, and to identify the most simple and sound model (18). After the final model was created, factor scores were calculated in MPlus. CFA confirmed validity of six EBDM organizational support latent factors with 23 of the 27 items: EBDM awareness, EBDM capacity, resources for EBDM, evaluation capacity, EBDM climate cultivation, and partnerships (Table 1) (18). Evaluation capacity was defined here as the health department's readiness to plan for evaluation, conduct evaluation, use findings to improve programs, and share findings. Table 1 shows the underlying items of the evaluation capacity factor.

**Table 1:** Evidence-based decision making (EBDM) support factors and items^a^.

**Factor**	**Item wording^b^**
Awareness of EBDM (3 items)	1. I am provided the time to identify evidence-based programs and practices.
	2.My direct supervisor recognizes the value of management practices that facilitate EBDM.
	3.My work group/division offers employees opportunities to attend EBDM trainings.
Capacity for EBDM (7 items)	1.I use EBDM in my work.
	2.My direct supervisor expects me to use EBDM.
	3.My performance is partially evaluated on how well I use EBDM in my work.
	4.My work group/division currently has the resources (e.g., staff, facilities, partners) to support application of EBDM.
	5.The staff in my work group/division has the necessary skills to carry out EBDM.
	6.The majority of my work group/division's external partners support use of EBDM.
	7.Top leadership in my agency encourages use of EBDM.
Resource availability (3 items)	1.Informational resources (e.g., academic journals, guidelines, and toolkits) are available to my work group/division to promote the use of EBDM.
	2.My work group/division engages a diverse external network of partners that share resources to facilitate EBDM.
	3.Stable funding is available for EBDM.
Evaluation capacity (3 items)	1. My work group/division plans for evaluation of interventions prior to implementation.
	2.My work group/division uses evaluation data to monitor and improve interventions.
	3. My work group/division distributes intervention evaluation findings to other organizations that can use our findings.
EBDM climate cultivation (3 items)	1. Information is widely shared in my work group/division so that everyone who makes decisions has access to all available knowledge.
	2. My agency is committed to hiring people with relevant training or experience in public health core disciplines (e.g., epidemiology, health education, environmental health).
	3.My agency has a culture that supports the processes necessary for EBDM.
Partnerships to support EBDM (3 items)	1. It is important to my agency to have partners who share resources (money, staff time, space, materials).
	2.It is important to my agency to have partners in healthcare to address population health issues.
	3.It is important to my agency to have partners in other sectors (outside of health) to address population health issues.

a *Factors derived through confirmatory factor analyses (18)*.

b *7-point Likert response options for each item, with 1 = strongly disagree to 7 = strongly agree*.

#### LHD Characteristics

Governance, jurisdiction population size, and dichotomous urbanization (urban or rural) measures from the 2016 NACCHO Profile Survey were obtained as potential control variables. LHD governance included two variables: presence of a local health board yes or no; and a categorical variable indicating state, local, or shared state, and local governance.

### Statistical Analysis

Survey data were cleaned and recoded in SPSS 24. Accreditation status was recoded into three levels: 0 = not preparing (answered no); 1 = preparing (not PHAB accredited and answered recently applied or preparing to apply); and 2 = PHAB-accredited at time of survey. Twenty-six LHDs were excluded from accreditation recoding (22 answered “unsure” and 4 left the item blank). Jurisdiction population size was recoded into three categories: <50,000, 50,000 to <200,000, and ≥ 200,000. CFA factor scores for each of the six organizational support factors were recoded into tertiles (18). There was one participant per LHD and so there was one LHD factor score for each of the six factors. A dichotomous dependent variable was then created for each factor, as the highest tertile (coded 1) vs. the lower two tertiles (coded 0) for each LHD. Bivariate logistic regression modeling was conducted in SAS 9.4 with each of the six EBDM support factors as the dependent variable in separate models, and three-level accreditation status as the independent variable of interest. Then, each of the six logistic regression models was adjusted for LHD governance (state, local, or shared state and local governance; presence of a local health board) and jurisdiction population size. We adjusted for LHD governance because LHDs were more likely to be accredited if they were governed by their state health department, and less likely to be accredited if they had a local board of health or were locally governed by any local entity (Table 2). No further adjustment was needed for rurality after adjusting for LHD jurisdiction population size, so the rurality variable was not in the final models.

**Table 2:** Local health department characteristics, by accreditation status, from a 2017 sample of U.S. local health department chronic disease prevention directors.

**Characteristic**	**Overall *N* = 350 %**	**Accreditation Status^a^ (*n* = 350)**			**Chi- square *P*- Value**
		**Accredited (confirmed) (*n* = 106) %**	**Preparing for accreditation (*n* = 138) %**	**Not preparing to apply (*n* =106) %**	
Jurisdiction population size					<0.001
<50,000	30.6	53.8	26.8	12.3	
50,000–199,000	34.3	31.1	38.4	32.1	
≥ 200,000	35.1	56.6	34.8	14.2	
Rural	45.1	30.2	39.9	67.0	<0.001
Local board of health	72.5	58.7	77.8	79.2	0.001
Governance structure					<0.001
Locally governed	76.6	54.7	83.3	89.6	
State governed	13.7	25.5	8.7	8.5	
Shared state/local governance	9.7	19.8	8.0	1.9	

a *Accreditation status*.

*Accredited, Accredited at time of survey confirmed per Public Health Accreditation Board list; Preparing for accreditation, Self-reported the health department recently applied for accreditation or was preparing to apply; Not preparing to apply, Self-reported the health department was not preparing to apply*.

## Results

The lead chronic disease control employee from 376 LHDs completed the survey, of 579 invited (65% response). There was one participant per LHD. Twenty-six (6.9%) participants answered accreditation preparation as unsure (*n* = 22) or left the item blank (*n* = 4) and were excluded from analyses (none were PHAB-accredited at time of survey). Of the 350 LHDs with usable accreditation information, 106 (30.3%) were PHAB-accredited at the time of the survey, 138 (39.4%) reported preparing for accreditation, and 106 (30.3%) reported not preparing (Table 2). Bivariate descriptive analyses showed that LHDs in rural jurisdictions were less likely to be accredited and more likely to report not preparing for accreditation than other LHDs (*p* < 0.001). State governance (*p* < 0.001), having a local board of health (*p* = 0.001), and jurisdiction population size ≥200,000 were positively associated with PHAB accreditation.

Table 3 shows logistic regression results of testing associations of accreditation status at time of survey (independent variable of interest) with each perceived EBDM support factor as the dependent variable in separate models. LHDs that reported preparing for accreditation showed a pattern of non-significant but higher perceived EBDM awareness and climate cultivation compared to those not preparing for accreditation. In models adjusted for governance and jurisdiction population size, PHAB-accredited LHDs were more likely to report higher EBDM capacity, EBDM resource availability, and evaluation capacity than those that reported not preparing for accreditation.

**Table 3:** Odds ratios of highest tertile of evidence-based decision making (EBDM) factors (dependent variables) by accreditation status in separate logistic regression models among U.S. local health departments, 2017 (*n* = 350).

**Dependent variables^a^ (EBDM organizational support factors)**	**Accreditation status^b^**	**Bivariate models**			**Models adjusted for LHD governance and jurisdiction population size^c^**		
		**OR**	**(95% CI)**	***P***	**Adj. OR**	**(95% CI)**	***P***
Awareness of EBDM	Not preparing	Ref.			Ref.		
	Preparing	1.44	(0.82, 2.54)	0.21	1.16	(0.63, 2.13)	0.64
	Accredited	**2.18**	**(1.21, 3.93)**	**0.009**	1.72	(0.85, 3.48)	0.13
EBDM capacity	Not preparing	Ref.			Ref.		
	Preparing	1.60	(0.90, 2.85)	0.11	1.19	(0.64, 2.23)	0.58
	Accredited	**2.72**	**(1.50, 4.93)**	**0.001**	**2.12**	**(1.04, 4.32)**	**0.04**
Resource availability	Not preparing	Ref.			Ref.		
	Preparing	**2.02**	**(1.12, 3.67)**	**0.02**	1.69	(0.90, 3.17)	0.10
	Accredited	**2.66**	**(1.43, 4.92)**	**0.001**	**2.24**	**(1.08, 4.57)**	**0.03**
Evaluation capacity	Not preparing	Ref.			Ref.		
	Preparing	1.60	(0.90, 2.85)	0.11	1.42	(0.77, 2.60)	0.26
	Accredited	**2.42**	**(1.34, 4.40)**	**0.004**	**2.26**	**(1.12, 4.56)**	**0.02**
Climate cultivation	Not preparing	Ref.			Ref.		
	Preparing	1.31	(0.76, 2.27)	0.34	1.21	(0.67, 2.18)	0.53
	Accredited	1.36	(0.76, 2.43)	0.30	1.37	(0.68, 2.75)	0.38
Partnerships that support EBDM	Not preparing	Ref.			Ref.		
	Preparing	1.31	(0.76, 2.26)	0.32	1.23	(0.70, 2.17)	0.47
	Accredited	1.19	(0.67, 2.12)	0.56	1.15	(0.59, 2.27)	0.68

*EBDM, Evidence-based decision making; LHD, Local health department; Bold, Statistically significant (p ≤ 0.05)*.

a *Each dependent variable was the highest tertile of the LHD confirmatory factor analysis scores (1 score per LHD) vs. the lower two tertiles. Each factor included 3–7 self-report items in 7-point Likert scales on perceived organizational supports for EBDM (18)*.

b *Accreditation status: Accredited, Accredited at time of survey confirmed per Public Health Accreditation Board list; Preparing for accreditation, Self-reported the health department recently applied for accreditation or was preparing to apply; Not preparing, Self-reported the health department was not preparing to apply for accreditation*.

c *Models were adjusted for presence of a local board of health, state health department governance (locally governed, state governed, shared state/local governance) and jurisdiction population size (<50,000, 50,000 to <200,000, ≥200,000)*.

## Discussion

PHAB accreditation is associated with higher perceived EBDM capacity, availability of resources for EBDM, and evaluation capacity among LHDs in this sample when controlling for governance and jurisdiction population size. Other EBDM support factors (awareness, climate cultivation) showed a consistent pattern of higher perceived supports among accredited LHDs than LHDs preparing for accreditation, which were higher than LHDs that reported not preparing, although the differences were smaller and not statistically significant in adjusted models.

The observed pattern of higher perceived health department performance among accredited health departments is consistent with the literature (12, 13). It is encouraging that accredited LHDs reported higher perceived EBDM capacity in the present study, given the PHAB emphasis on workforce development in Domain 8 and throughout the accreditation process (21–24). Domain 4 of the PHAB standards calls for community engagement to identify and address community needs (2). Although there were no significant associations between accreditation and partnerships that support EBDM in the present study, other authors found enhanced collaboration among accredited health departments (15, 25).

Small jurisdiction population size and rurality are known to be associated with lower likelihood of health department accreditation or preparations for accreditation (26). Health departments with fewer employees conduct community health assessments and planning but find it difficult to address all the accreditation standards (27). Maintaining a workforce with the needed skills for EBDM, quality improvement (QI), and cross-sector collaboration is challenging, especially in agencies with limited workforce development infrastructure (27).

Study findings should be considered in light of the following limitations: use of self-report, limited representativeness, cross-sectional study design, and inadequate statistical power for certain sub-group analyses. Accreditation involves the LHD as a whole, and all departments within the LHD need to be applying EBDM. In these analyses, representativeness is limited because the survey sample from which we drew the data had a chronic disease prevention focus. For the larger study, we randomly selected LHDs that had reported in the 2016 NACCHO Profile survey that they provided diabetes screening, body mass index screening, or population-based physical activity or nutrition activities to meet the chronic disease focus of the larger study. Representativeness is also limited by having surveyed only the person in charge of community-based chronic disease prevention, where identifiable, or otherwise the LHD director. In larger LHDs, our findings may apply only to the unit/s that conduct community-based chronic disease prevention initiatives rather than to the LHD as a whole. From the present analyses, it is not possible to discern whether EBDM supports in a LHD are limited to or extend beyond the unit/s responsible for community-based chronic disease prevention and control. Social desirability bias may lead survey participants to overreport EBDM supports and there may be differential reporting by leaders compared with frontline staff (28, 29). Survey responses are the responding individual's views and may not represent the views of the entire health department. Respondents may have interpreted item wording differently.

Organizational supports for EBDM are modifiable but are complex and take time and concerted effort to change (6). A randomized cluster trial testing EBDM capacity enhancement through training and technical assistance with state health department chronic disease units found that skills and perceived access to evidence and skilled staff significantly increased among intervention sites compared to control sites, while other supports did not show an intervention effect (30). Promotion and funding support from national agencies have fueled changes in performance management, QI, and organizational supports for EBDM that facilitate the growing national accreditation movement (31, 32).

PHAB accreditation standards are intended to stimulate public health workforce development to ensure skilled public health staff, QI, performance management, evidence-informed decision making, and evidence-based practice that will lead to improved population health (1, 2). In this sample, PHAB-accredited LHDs reported higher capacity for EBDM, availability of resources for EBDM, and evaluation capacity than LHDs not preparing for accreditation. Survey findings support the premise that the national voluntary accreditation movement is linked with improved health department performance. PHAB standards may help motivate organizational supports for EBDM.

## Data Availability Statement

The datasets generated for this study are available upon request to the corresponding author.

## Ethics Statement

The studies involving human participants were reviewed and approved by the Human Research Protection Office, Washington University in St. Louis. The participants provided their written informed consent to participate in this study.

## Author Contributions

RB, RT, RP, and PA: conceptualization and design and survey instrument development. PA and SM: statistical analysis. PA, SM, RT, RP, MR, and RB: review of analyses. PA, RP, and RB: writing. PA, RB, SM, RP, RT, and MR: manuscript content revisions. All authors read and approved the final manuscript.

### Conflict of Interest

The authors declare that the research was conducted in the absence of any commercial or financial relationships that could be construed as a potential conflict of interest.
